# 0102. *Ex vivo* effects of hyperglycemia on phenotype and production of reactive oxygen species by the nadph oxidase of human immune cells in acute inflammatory response

**DOI:** 10.1186/2197-425X-2-S1-P13

**Published:** 2014-09-26

**Authors:** B Soyer, V Faivre, C Damoisel, A-C Lukaszewicz, D Payen

**Affiliations:** Hôpital Lariboisiere - APHP, Département d'Anesthésie Réanimation, Paris, France

## Introduction

Glucose is a major energy substrate for polymorphonuclears (PMNs) and monocytes (Monos). In acute stress, glucose was shown to be pro-inflammatory favoring the production of reactive oxygen species (ROS).

## Objectives

1- Demonstrate an increase in ROS production during moderate hyperglycemia by increased production of the NADPH oxidase through the pentose shunt pathway (PSP), the major source of NADPH.

2- Evaluate the phenotypic consequences on PMNs, Monos, and Lymphocytes (Lymphos).

## Methods

Whole blood from healthy volunteers (CCPSL UNT -No 13/SL/015) was incubated 90 minutes either in normoglycemia (NG) or hyperglycemia (HG, 12.5 mM glucose), in the presence of deoxyglucose (DOG, 12.5 mM) blocking glycolysis, or inhibitors of the PSP (Epi Androsterone & 6 Aminonicotinamide (EPI, 6AN)). For the all conditions: evaluation of overall ROS production (luminometry^1^: area under the curve (AUC) & slope of activation (Slope)) in resting cell condition & after stimulation by PMA-Ionomycin. Following phenotypic parameters were evaluated: HLA-DR expression on Monos (overall population, and subtypes CD16- and CD16+) and on B cells; expression of CD11b on Monos and PMNs; CTLA4 on T4/T8 Lymphos, and CD62L (marker of cell activation) on Monos, PMNs and Lymphos. Statistics: non-parametric tests.

## Results

### 1-ROS

In resting conditions: AUC of luminometry (increased production) (n = 19) and Slope (system reactivity) were higher in HG than in NG (p< 0.05), a difference that disappeared after PMA-Ionomycin stimulation. The inhibitors (DOG, EPI + 6AN) reduced the AUC and the Slope in NG and HG (p< 0.05 and p< 0.05).

### 2-Cell phenotype

HG did not change the cell expression of markers for Monos (total, CD16- and CD16+), PMNs, T4 and T8 Lymphos. Only in B cell HG increased HLA-DR expression (p< 0.05). The DOG condition decreased expression of HLA-DR, CD11b and CD62L on Monos and CD62L on PMNs (p< 0.05), with no effect on Lymphos.

## Conclusions

HG amplifies ROS production and NADPH oxidase reactivity via PSP pathway and NADPH synthesis, especially after stimulation in Monos. Absence of any impact of acute glycemic variation and of blocking glucose metabolism on T4, T8 Lymphos phenotype, strongly suggests that Lymphos do not use glucose as a major energetic substrate^2^. Except for B lympho, Mono and PMNs did not change their studied phenotype, with *ex vivo* non-significant effect on T Lymphos. Acute HG as glucose metabolic inhibitors changed ROS production but not innate immune cell phenotype.
